# Patterns and Timing of Sequential Transcatheter Valve Interventions After Index TAVR, M-TEER, and TMVR: A Nationwide Medicare Analysis

**DOI:** 10.1016/j.shj.2026.101072

**Published:** 2026-06-12

**Authors:** Nicolò Azzola Guicciardi, Jeffrey Popma, Martin B. Leon, David J. Cohen, Roberto Lorusso, Francesco Maisano, Michele De Bonis, Philipp von Stein, Jennifer von Stein, Maria Alu, Alexandra Popma, Juan F. Granada, Harun Kundi

**Affiliations:** a Cardiovascular Research Foundation, New York, New York, USA; b Department of Cardiac Surgery, IRCCS San Raffaele Scientific Institute, Vita-Salute San Raffaele University, Milan, Italy; c Department of Cardiology, Beth Israel Deaconess Medical Center, Boston, Massachusetts, USA; d Department of Cardiology, Columbia-Presbyterian Medical Center, New York, New York, USA; e Department of Cardiology, St. Francis Hospital and Heart Center, Roslyn, New York, USA; f Department of Cardiology, Heart & Vascular Centre, Maastricht University, Maastricht, Netherlands; g Department of Cardiology, Heart Center, Faculty of Medicine, University of Cologne, Cologne, Germany

**Keywords:** Epidemiology, Medicare, Multivalvular disease, Transcatheter valve interventions

## Abstract

• 5 yr downstream transcatheter valve interventions: 0.86% transcatheter aortic valve replacement (TAVR), 2.69% mitral transcatheter edge-to-edge repair (M-TEER), and 4.48% transcatheter mitral valve replacement (TMVR).
• Main sequences: TAVR→M-TEER, M-TEER→TAVR, and TMVR→TAVR.
• Median time: 229 d (TAVR), 300 d (M-TEER), and 483 d (TMVR).

The growing prevalence of multiple valvular heart disease in an aging population,^1^ together with the expansion of transcatheter therapies across valve positions, has shifted the management of valvular heart disease from isolated procedures toward a more longitudinal, multivalve treatment strategy.^2^ However, real-world data describing how frequently these multivalve interventions are performed and sequenced remain limited. We therefore characterized the frequency, sequencing, and timing of additional transcatheter valve interventions (TVIs) following index transcatheter aortic valve replacement (TAVR), mitral transcatheter edge-to-edge repair (M-TEER), or transcatheter mitral valve replacement (TMVR) in a nationwide Medicare cohort.

We performed a retrospective cohort study using 100% Medicare Fee-for-Service claims. This study was conducted in compliance with all applicable Medicare regulations, federal and state laws, and ethical guidelines governing the use of Medicare data. Patients undergoing index TAVR, M-TEER, or TMVR between January 1, 2017, and December 31, 2023, were identified from the Medicare Provider Analysis and Review file using International Classification of Diseases, 10th Revision (ICD-10), Procedure Coding System codes, as previously described.^3^^,^^4^ The index procedure was defined as the first qualifying TVIs during the study period. Patients were subsequently followed for additional TVIs involving a different valve from the index procedure (TAVR, M-TEER, or TMVR), identified using ICD-10-Procedure Coding System codes. The presence and severity of concomitant valvular heart disease were not reported at time of index TVI. Timing from the index procedure to the first additional intervention was summarized using the median and interquartile range. Follow-up continued until death, end of the study period, or loss of Medicare Fee-for-Service enrollment. The 5-year cumulative incidence of downstream intervention was estimated using a competing-risk framework, with death treated as a competing event.

Baseline demographic characteristics were obtained from Medicare enrollment records. Comorbidities were identified from diagnosis codes marked as present on admission during the index hospitalization or from inpatient claims in the 1 year preceding the index procedure. Frailty was assessed using the Hospital Frailty Risk Score, a validated claims-based measure derived from 109 ICD-10 diagnosis codes, and patients with a score >5 were classified as frail.^5^ Mortality was ascertained from the Medicare Master Beneficiary Summary File through December 31, 2023. Among patients undergoing a first subsequent transcatheter intervention, 1-year mortality from the time of the downstream procedure was estimated using the Kaplan–Meier method.

A total of 527,976 beneficiaries underwent an index TVI during the study period, including 461,272 undergoing TAVR, 59,782 undergoing M-TEER, and 6922 undergoing TMVR. The mean age was similar in the TAVR and M-TEER cohorts (80.1 ± 7.5 and 80.0 ± 7.3 years, respectively) and slightly lower in the TMVR cohort (78.2 ± 6.5 years), whereas female representation ranged from 43.4% to 55.2%. Clinical risk burden was substantial across all cohorts, with heart failure present in 74.1%, 90.1%, and 92.1% of patients and frailty (Hospital Frailty Risk Score >5) in 33.2%, 45.7%, and 45.3% of the TAVR, M-TEER, and TMVR cohorts, respectively (Table 1). At the time of the index procedure, concomitant valve disease included mitral regurgitation and tricuspid regurgitation (TR) in the TAVR cohort (5.3% and 2.6%, respectively), TR and aortic stenosis in the M-TEER cohort (11.7% and 3.2%), and TR and aortic stenosis in the TMVR cohort (9.5% and 4.8%). The median follow-up duration for the overall cohort was 2.8 years (interquartile range [IQR] 1.4–4.6).

**Table 1:** Index cohort characteristics and first downstream transcatheter valve interventions with timing and Kaplan–Meier–estimated 1-year mortality

Index procedure	Age, mean ± SD, female sex (%)	Key comorbidities (%)	Any downstream intervention, 5 y CIF (95% CI)	Type of first subsequent intervention after index procedure n (%)	Median time to first subsequent intervention, days (IQR)	KM–estimated 1-y mortality after first subsequent intervention (%)
TAVR n = 461,272	80.1 ± 7.5, 55.2%	HF: 74.1% CKD: 36.5% COPD: 28.2% Frail: 33.2%	0.86% (95% CI, 0.83%–0.90%)	M-TEER: 2289 (0.5%) TMVR: 481 (0.1%) T-TEER: 113 (0.02%)	229 (84–642)	M-TEER: 25.9% TMVR: 27.0% T-TEER: 27.1%
M-TEER n = 59,782	80.0 ± 7.3, 53.5%	HF: 90.1% CKD: 46.9% COPD: 31.9% Frail: 45.7%	2.69% (95% CI, 2.53%–2.87%)	TAVR: 869 (1.45%) T-TEER: 249 (0.42%)	300 (99–702)	TAVR: 22.2% T-TEER: 27.7%
TMVR n = 6922	78.2 ± 6.5, 43.4%	HF: 92.1% CKD: 45.4% COPD: 36.1% Frail: 45.3%	4.48% (95% CI, 3.82%–5.26%)	TAVR: 153 (2.21%) T-TEER: 20 (0.29%)	483 (116–994)	TAVR: 12.8% T-TEER: 38.3%

Values are presented as n (%) unless otherwise indicated. Frailty was defined as Hospital Frailty Risk Score >5. One-year mortality was estimated using the Kaplan–Meier method from the time of the downstream intervention.

Abbreviations: CIF, cumulative incidence function; CKD, chronic kidney disease; COPD, chronic obstructive pulmonary disease; HF, heart failure; IQR, interquartile range; KM, Kaplan–Meier; M-TEER, mitral transcatheter edge-to-edge repair; TAVR, transcatheter aortic valve replacement; TMVR, transcatheter mitral valve replacement; T-TEER, tricuspid transcatheter edge-to-edge repair.

Additional TVIs remained uncommon across all index procedures, with 5-year cumulative incidence function estimates of 0.86% (95% CI, 0.83%–0.90%) after TAVR, 2.69% (95% CI, 2.53%–2.87%) after M-TEER, and 4.48% (95% CI, 3.82%–5.26%) after TMVR. Despite their low overall frequency, observed downstream intervention pathways demonstrated consistent directional patterns across cohorts (Figure 1). After TAVR, the most common observed first subsequent intervention was M-TEER (0.50%), followed by TMVR (0.10%), whereas tricuspid intervention with T-TEER was rare (0.02%). After M-TEER, the most common observed subsequent intervention was TAVR (1.45%), followed by T-TEER (0.42%). Similarly, after TMVR, TAVR was the dominant observed subsequent intervention (2.21%), followed by T-TEER (0.29%). We also assessed multistep pathways, which were consistently rare across all cohorts (<0.1%) (Figure 1).

**Figure 1:**
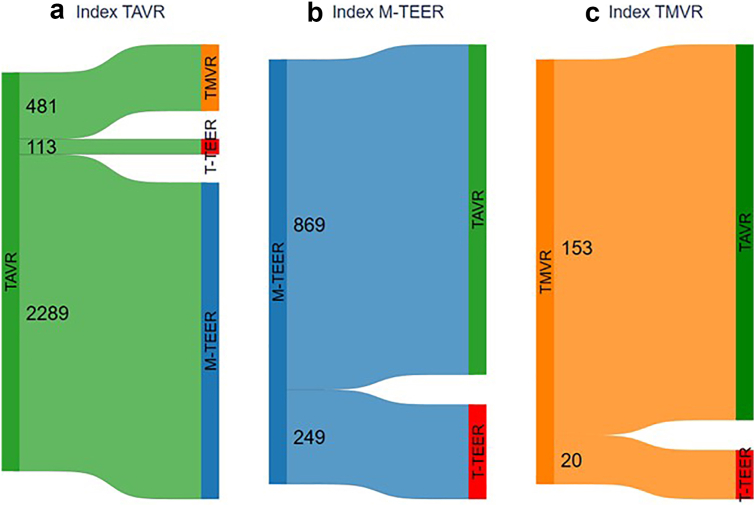
**First downstream transcatheter valve intervention following index TAVR, M-TEER, and TMVR.** Three-panel Sankey diagrams display the first downstream transcatheter valve intervention after each index procedure. Flow width is proportional to pathway frequency Abbreviations: M-TEER, mitral transcatheter edge-to-edge repair; TAVR, transcatheter aortic valve replacement; TMVR, transcatheter mitral valve replacement; T-TEER, tricuspid transcatheter edge-to-edge repair.

The temporal distribution of staged interventions also differed by index procedure. After TAVR, the median time to first downstream intervention was 229 days (IQR, 84–642), with most events occurring within the first year. After M-TEER, the median time to first downstream intervention was 300 days (IQR, 99–702), again with a predominance of early events. In contrast, downstream interventions after TMVR occurred later, with a median time to first downstream intervention of 483 days (IQR, 116–994) and a substantial proportion of downstream TAVR procedures occurring beyond 2 years (Table 1).

Among patients undergoing a downstream intervention, Kaplan–Meier–estimated 1-year mortality was generally similar according to the type of subsequent procedure. In the TAVR cohort, 1-year mortality after downstream M-TEER, TMVR, and T-TEER was 25.9%, 27.0%, and 27.1%, respectively. In the M-TEER cohort, 1-year mortality after downstream TAVR and T-TEER was 22.2% and 27.7%, respectively. The main exception was observed in the TMVR cohort, in which 1-year mortality was 12.8% after downstream TAVR and 38.3% after downstream T-TEER (Table 1).

Several observations emerge from this analysis. First, multiple TVIs were infrequent in contemporary practice, even in this large nationwide cohort. Second, the observed downstream pathways followed consistent directional patterns that may reflect underlying disease progression and hemodynamic interactions between valve lesions after treatment of the dominant valve pathology.^6^ Third, most pathways consisted of a single additional intervention, whereas multistep transcatheter sequences were rare.

Although these findings should not be interpreted as evidence of treatment effectiveness, they suggest that sequential transcatheter intervention can occur in selected patients as part of longitudinal valve care. From a clinical perspective, these data may help inform anticipatory decision-making at the time of the index procedure, particularly in patients with multivalve disease.

The temporal distribution of further interventions may also have implications for follow-up strategies. The clustering of events within the first year after TAVR and M-TEER suggests that early surveillance may be particularly relevant in these cohorts, whereas the later pattern observed after TMVR supports continued longer-term monitoring.

This study has several limitations. First, the use of administrative claims data limits the ability to capture detailed clinical and imaging information, including valve disease severity and procedural indications. Conversely, the frequency of subsequent percutaneous interventions in this study may underestimate the frequency among patients with truly severe concomitant multiple valve disease at baseline. Second, identification of subsequent interventions relies on coding accuracy and may not capture all clinically relevant transitions. Third, postdownstream mortality estimates are descriptive and reflect a selected population undergoing downstream intervention, precluding causal inference. Finally, procedural complications and short-term safety outcomes were not evaluated.

In conclusion, subsequent TVIs following TAVR, M-TEER, and TMVR were uncommon but followed consistent directional patterns. These findings support the concept that sequential transcatheter strategies may be feasible in selected patients as part of longitudinal valve care.

## Funding

The authors have no funding to report.

## Ethics statement

This study was conducted in compliance with all applicable Medicare regulations, federal and state laws, and ethical guidelines governing the use of Medicare data.

## Review Statement

The review of this manuscript was managed by Guest Editor Harold Dauerman, MD.

## Disclosure Statement

Jeffrey Popma is a former employee of and has nonvested equity in 10.13039/100004374Medtronic. Martin B. Leon has received institutional research support from 10.13039/100006520Edwards Lifesciences, 10.13039/100004374Medtronic, 10.13039/100008497Boston Scientific, and 10.13039/100001316Abbott Laboratories and is a consultant or advisory board member for Foldax, Anteris, JenaValve, Medinol, SoloPace, and Bain Capital. David J. Cohen has received institutional research support from 10.13039/100001316Abbott Laboratories, 10.13039/100006520Edwards Lifesciences, 10.13039/100008497Boston Scientific, Philips, JenaValve, and Zoll Medical and has received consulting income from 10.13039/100001316Abbott Laboratories, 10.13039/100006520Edwards Lifesciences, 10.13039/100008497Boston Scientific, 10.13039/100004374Medtronic, and Elixir Medical. Juan F. Granada is the cofounder of Cephea Valve Technologies (10.13039/100001316Abbott Laboratories) and chief executive officer of the Cardiovascular Research Foundation.

The other authors have no conflicts to declare.
